# Case report: Management of generalized infection and draining tracts of the frontomaxillary region in a dog

**DOI:** 10.3389/fvets.2024.1343039

**Published:** 2024-02-16

**Authors:** Elias Wolfs, Ching Ching Shirley Kot, Natalia Vapniarsky, Boaz Arzi

**Affiliations:** ^1^Dentistry and Oromaxillofacial Surgery, Department of Surgical Sciences, School of Veterinary Medicine, University of Wisconsin-Madison, Madison, WI, United States; ^2^Dentistry and Oral Surgery Service, CityU Veterinary Medical Centre, Kowloon, Hong Kong SAR, China; ^3^Department of Pathology, Microbiology and Immunology, School of Veterinary Medicine, University of California, Davis, Davis, CA, United States; ^4^Department of Surgical and Radiological Sciences, University of California, Davis, Davis, CA, United States

**Keywords:** computed tomography, skull, axial pattern flap, fistula, draining tract

## Abstract

**Objective:**

This study aims to report the surgical and medical management of generalized chronic maxillofacial infection with multiple intra- and extraoral draining tracts in a dog.

**Case summary:**

A 6 years-old, male neutered pit bull terrier dog underwent a staged procedure. First, a diagnostic work-up including hematologic and biochemical analysis, conventional computed tomography (CT) with contrast of the skull, and a rhinoscopic evaluation of the draining tracts was performed. Samples were obtained for histopathological, microbial, and fungal testing. Second, a 4 week course of antimicrobials based on culture and sensitivity results was administered. Third, an extraoral approach to soft tissue reconstruction was accomplished as a first stage in the repair process. Finally, an intraoral approach to repair the oronasal fistulous draining tracts was performed. A 6 months follow-up skull CT revealed various stages of repair and remodeling and adequate soft tissue healing.

**Clinical relevance:**

A staged procedure is a suitable option to treat chronic and generalized frontal and maxillary infection with multiple intra- and extraoral fistulous draining tracts in dogs.

## 1 Introduction

Generalized infection with multiple draining tracts of the maxillofacial region is a relatively infrequent issue but has a major clinical impact that requires dogs to be examined by veterinarians. In part, this may be due to the abundant blood supply and collateral circulation of the craniofacial region in the dog (1), making it less prone to infection compared to other parts of the body. Invasion of bacteria originating from the oral microbiota, skin, or respiratory tract into the deeper tissues and the proximity of bone typically results in inflammation that leads to vascular changes that include but are not limited to vasodilation, thrombosis, endothelial damage, exudation of serum into the extravascular space, the release of free radicals, and the extravasation of polymorphonuclear cells (2). These changes, in turn, may lead to ischemic damage of the soft tissue and bone and may manifest as single or multiple areas of osteolysis and fistulation. The introduction of bacteria may result from trauma, bone surgery, bacteremia, or a contiguous infectious focus and is further influenced by diseases that affect the vascularity of bone and by systemic diseases that alter the innate host defenses (3). Examples of systemic conditions that decrease host defenses include diabetes, anemia, immunosuppression, and malnutrition (4–6). Causes of immunosuppression include but are not limited to chemotherapy, glucocorticoids, and cyclosporine treatment (medication-related) or an underlying disease process that may weaken the immune system, such as lymphoma and immune-mediated neutropenia. Radiation, malignancy, and medications such as bisphosphonates are examples that may contribute to decreased vascularity of bone, thus predisposing to infection and necrosis (3, 7, 8).

We report a successfully staged surgical repair of chronic maxillofacial infection caused by a multi-drug resistant coagulase-negative *Staphylococcus* spp. with osteolysis and multiple draining tracts in a 6 years-old male neutered pit bull terrier dog. The procedure was performed to resolve the clinical signs associated with the condition.

## 2 Clinical report

### 2.1 Case presentation and diagnostic investigations

A 6 years-old male neutered pit bull terrier was presented for chronic infection and multiple draining tracts of the craniofacial region. The clients reported that the clinical signs including open wounds on the face, a foul odor from the mouth, and hyporexia that started approximately 3 years earlier. The dog was treated at various first opinion practices by means of sedated explorations of the extra- and intraoral lesions, where foreign plant material was retrieved on multiple occasions. A combination of primary closure and placement of Penrose drains was attempted, samples were collected for microbial culture tests, and analgesia and antimicrobial therapy based on culture results were prescribed. Over the course of 3 years, the following antimicrobials were prescribed: amoxicillin/clavulanate, ampicillin, amikacin, cefovecin, cephalexin, minocycline, and orbifloxacin. Due to the progressively worsening nature of the lesions, size, and invasion into deeper tissues after attempted repairs, the dog was referred.

**Figure 1:**
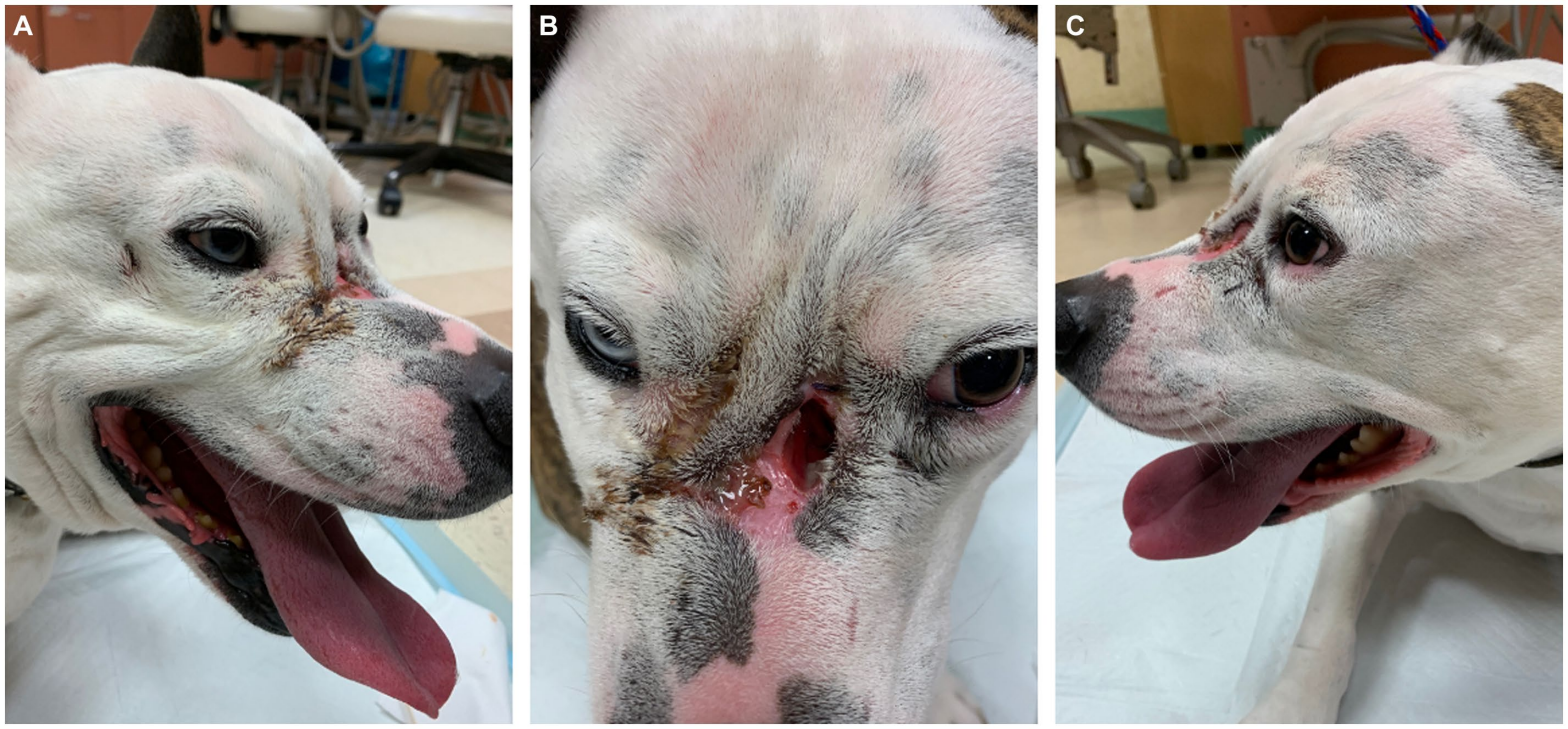
Note the obvious large mid-facial defect **(B)** accompanied by smaller defects **(A)** and **(C)** ventral to both eyes. All lesions were actively draining with mucopurulent and crusted discharge. Incidental findings of marked anisocoria and heterochromia iridum.

**Figure 2:**
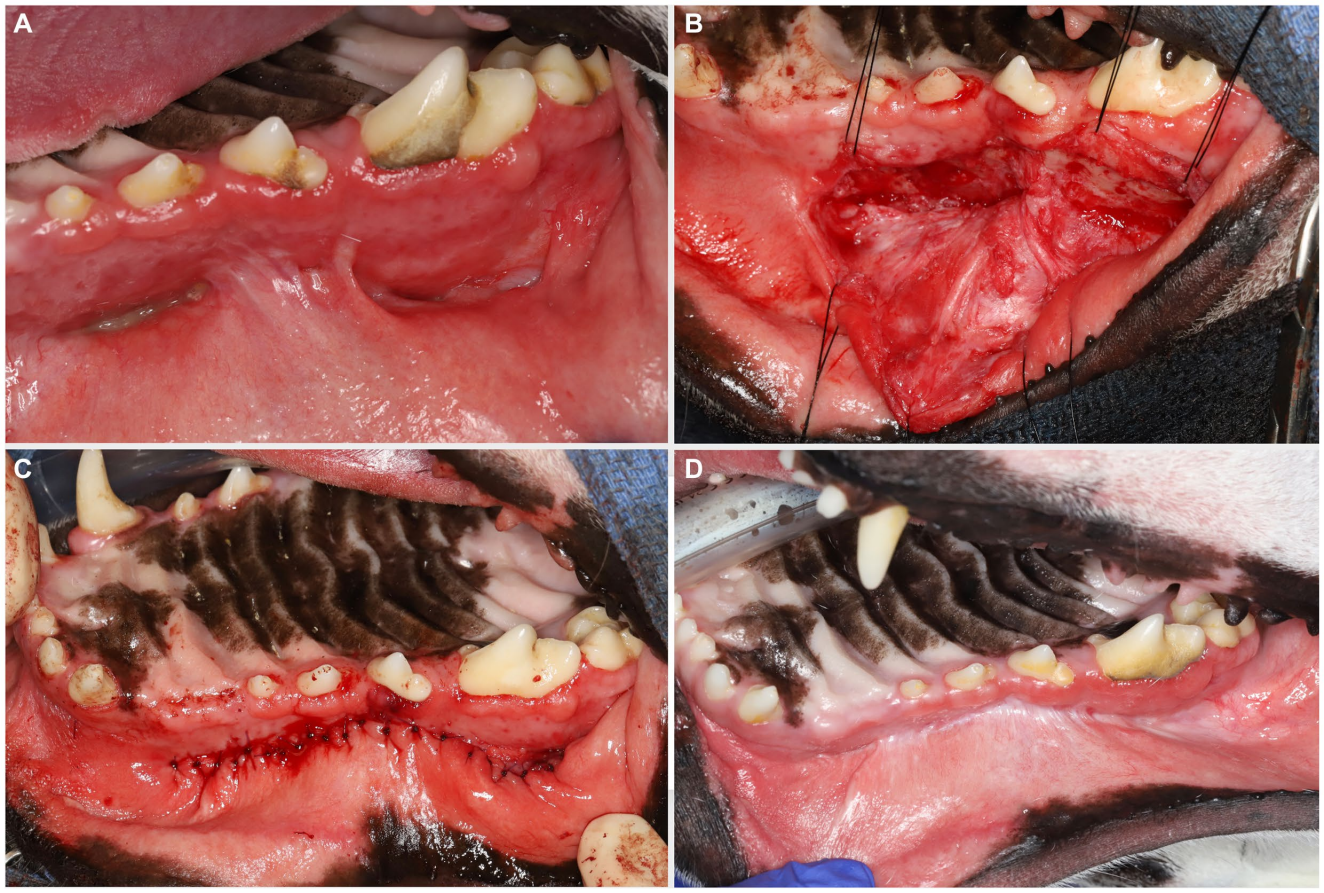
Large oronasal fistulas **(A)** were repaired at a second stage procedure. A large mucoperiosteal flap was elevated and manipulated using stay sutures **(B)**. Note that the infraorbital neurovascular bundle was left intact to preserve blood supply. After removing the epithelial lining of the fistulas, tension-free closure was achieved with single interrupted resorbable sutures **(C)**. A 6 months follow-up revealed excellent healing **(D)**.

**Figure 3:**
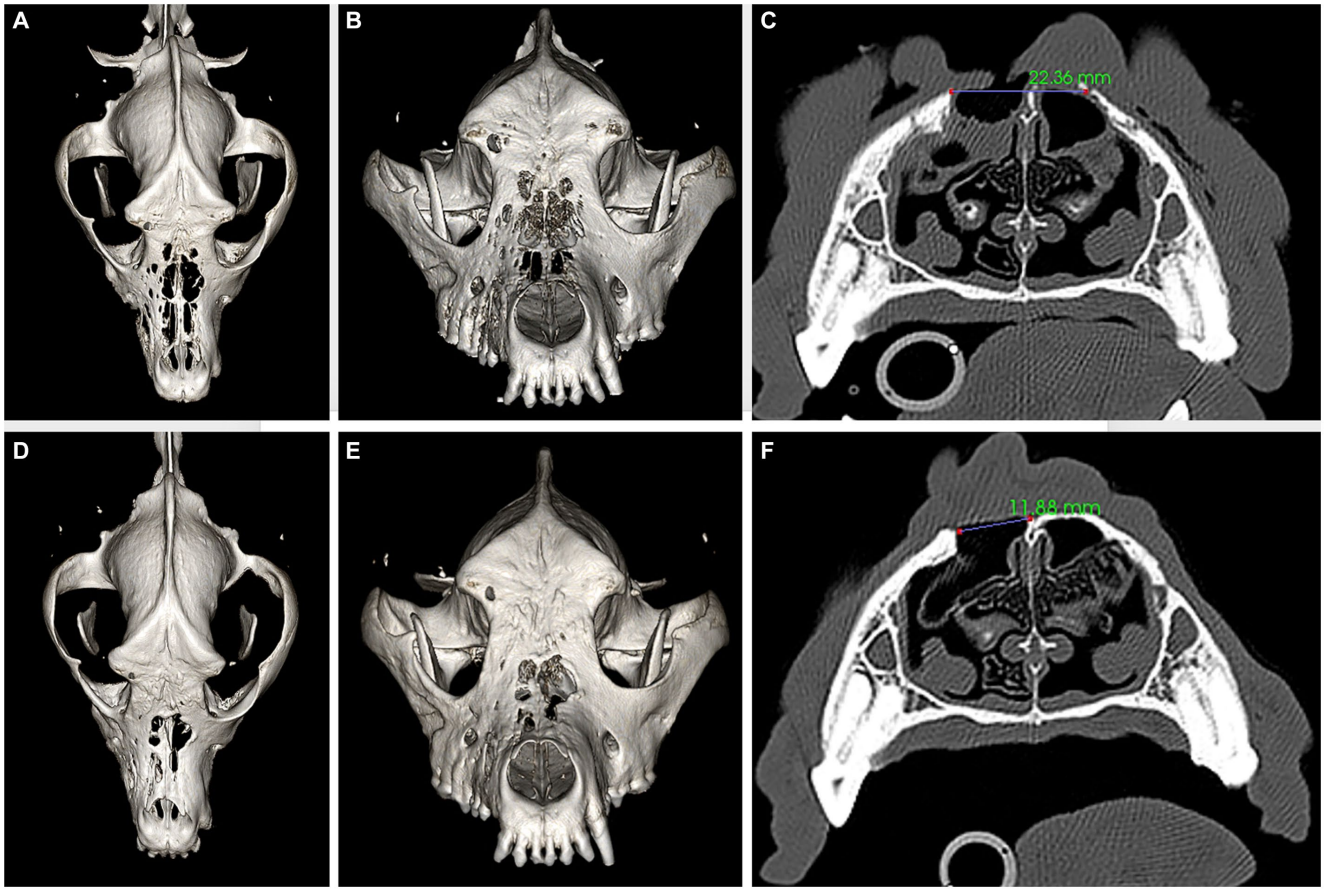
Panels **(A–C)** represent pre-operative CT, three-dimensional (3D) reconstruction **(A,B)**, and a transverse plane at the level of the fourth premolar tooth **(C)** images. Panels **(D–F)** represent 6 months postoperative CT, 3D reconstruction **(D,E)**, and a transverse plane at the level of the fourth premolar tooth **(F)** images. The large bone defects in the frontal, maxillary, and nasal bones **(A–C)** have reduced in size **(D–F)** after successful reconstruction, as demonstrated by the measurements provided in panels **(C)** and **(F)**.

On presentation, the dog appeared clinically stable. A large (20 × 20 mm) draining tract overlying the bridge of the nose between the eyes was observed (i.e., affecting the frontal, nasal, and maxillary bones). There were four additional cutaneous draining tracts noted approximately 4 × 4 mm ventral to the left eye, to the lateral side of the right eye, between the eyes, and on the medial aspect of the right eye (Figure 1). An intraoral examination revealed an oronasal fistula extending from the right maxillary canine tooth to the right maxillary fourth premolar tooth (Figure 2A). The right maxillary buccal frenulum remained intact. There was mucopurulent discharge and crusting surrounding the multiple draining tracts. There was bilateral mandibular lymphadenopathy. Incidental physical examination findings included cataracts with nuclear sclerosis, iris atrophy, heterochromia iridum, and anisocoria. The hematologic assessment revealed leukocytosis (14,900/μL; reference interval 6,000–13,000/μL) characterized by mild neutrophilia (11,507/μL; reference interval 3,000–10,500/μL). The serum biochemical values were within reference ranges. The dog underwent general anesthesia for conventional computed tomography (CT) (HiSpeed FX/i or LightSpeed16, GE Healthcare, Waukesha, WI) of the skull with and without contrast (880 mg/kg, IV, iopamidol). Computed tomographic findings included multifocal to coalescing lucent osseous lesions predominantly affecting the cortical bone of the right rostral maxilla (Figures 3A,B). There was a resultant large draining tract affecting the nasal, frontal, and maxillary bones that communicated with the cutaneous surface (Figure 3C). Differential diagnosis included a neoplastic process (i.e., squamous cell carcinoma, osteosarcoma, and round cell tumor) or an infectious etiology (i.e., coccidiomycosis, aspergillosis, and bacterial infection); a component of osteomyelitis associated with the nasal fistulae was anticipated. There was a mildly contrast-enhancing soft tissue structure in the right frontal sinus (which could reflect a neoplastic mass or granuloma). The right mandibular and medial retropharyngeal lymph nodes were enlarged (it was unknown whether they were reactive or metastatic). Finally, severe right temporomandibular joint osteoarthritis was observed, which may be secondary to prior trauma or infection of the joint. The CT was followed by rhinoscopy and was performed using a combination of intra- and extraoral (trans-fistulous) approaches. The findings confirmed most of the previously described osseous and soft tissue lesions noted on CT. No gross lesions consistent with fungal plaques were found. Following rhinoscopy, samples of the lesions were surgically harvested utilizing rongeurs and iris scissors for histopathological evaluation, as well as microbial and fungal testing. A Stent bandage was placed and kept in place for 3 days. The dog was discharged with analgesia (carprofen 2.2 mg/kg, PO, q12h and gabapentin 10 mg/kg, PO, q12h) and a broad-spectrum antimicrobial amoxicillin/clavulanate (13.75 mg/kg, PO, q12h).

**Figure 4:**
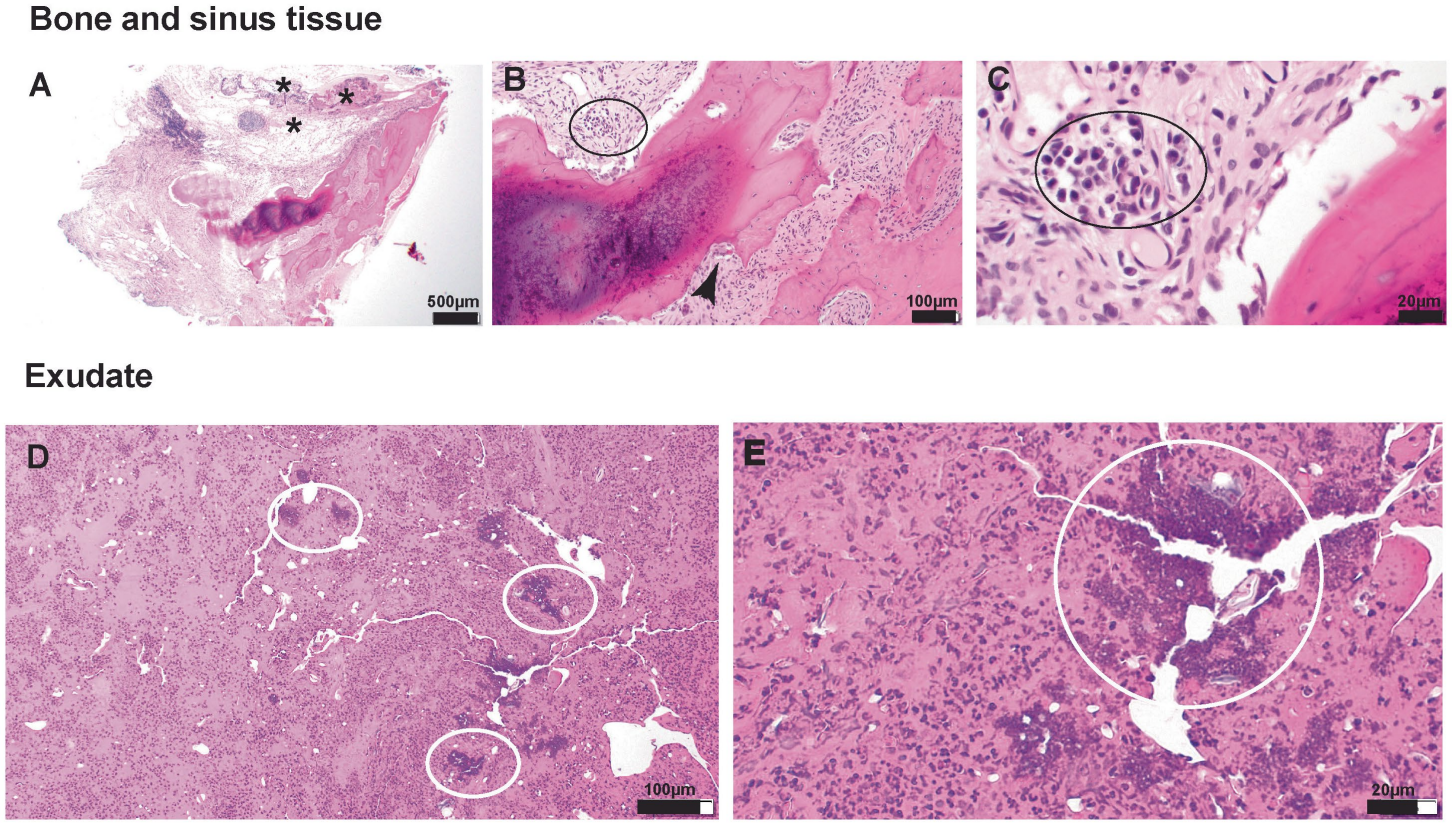
**(A–E)** Photomicrographs of tissue submitted for histopathological evaluation. **(A)**—20× magnification of the section of the bone with associated respiratory epithelium (presumes sinus lining). The subepithelial tissue is markedly altered and thickened by edema, fibrin exudation, and inflammatory cellular infiltration (asterisk). **(B)**—100× magnification of the tissue adjacent to the bone spicule. The inflammatory infiltrate comprises lymphocytes and plasma cells (black oval). In addition, the resorption lacuna occupied by osteoclast is shown (black arrowhead), indicative of bone resorption/modeling. **(C)**—400× magnification of the inflammatory infiltrate shown in **(B)**. **(D)**—100× magnification of an exudate composed of protein-rich (hot pink background) fluid suspending hundreds of polymorphonuclear cells (neutrophils primarily) and bacterial colonies (white ovals). **(E)**—400× magnification of bacterial cocci colonies shown in **(D)**.

The biopsy sample submitted for histopathological evaluation consisted of exudate with crust and an osseous fragment associated with soft tissue (Figure 4). The bone tissue was a fragment of lamellar bone (mature bone) rimmed multifocally by woven bone (immature bone) (Figure 4A). Multifocally resorption lacunae occupied by multinucleated osteoclasts were noted in the absence of osteoblasts, indicative of bone remodeling (Figure 4B). Infection of the bone medullary cavities was not noted. The crust and exudate suspended large numbers of intact and degenerate neutrophils admixed with bacterial cocci colonies and birefringent foreign material resembling plant and pollen (Figures 4C–E).

Soft tissue associated with the bone fragment included loose fibrous connective tissue infiltrated by scattered lymphocytes and plasma cells and round clusters of lymphocytes (lymphofollicular hyperplasia). This fibrous connective tissue was lined by primarily pseudostratified columnar ciliated epithelium that was multifocally substituted by squamous epithelial lining, indicative of squamous metaplasia (Figure 4).

The microbial culture and susceptibility results revealed the presence of multi-drug resistant coagulase-negative *Staphylococcus* spp., and therefore, the antimicrobial treatment was changed to enrofloxacin (10 mg/kg, PO, q12h) for 4 weeks, as it was identified as the susceptible antimicrobial of choice.

### 2.2 Treatment and clinical outcome

**Figure 5:**
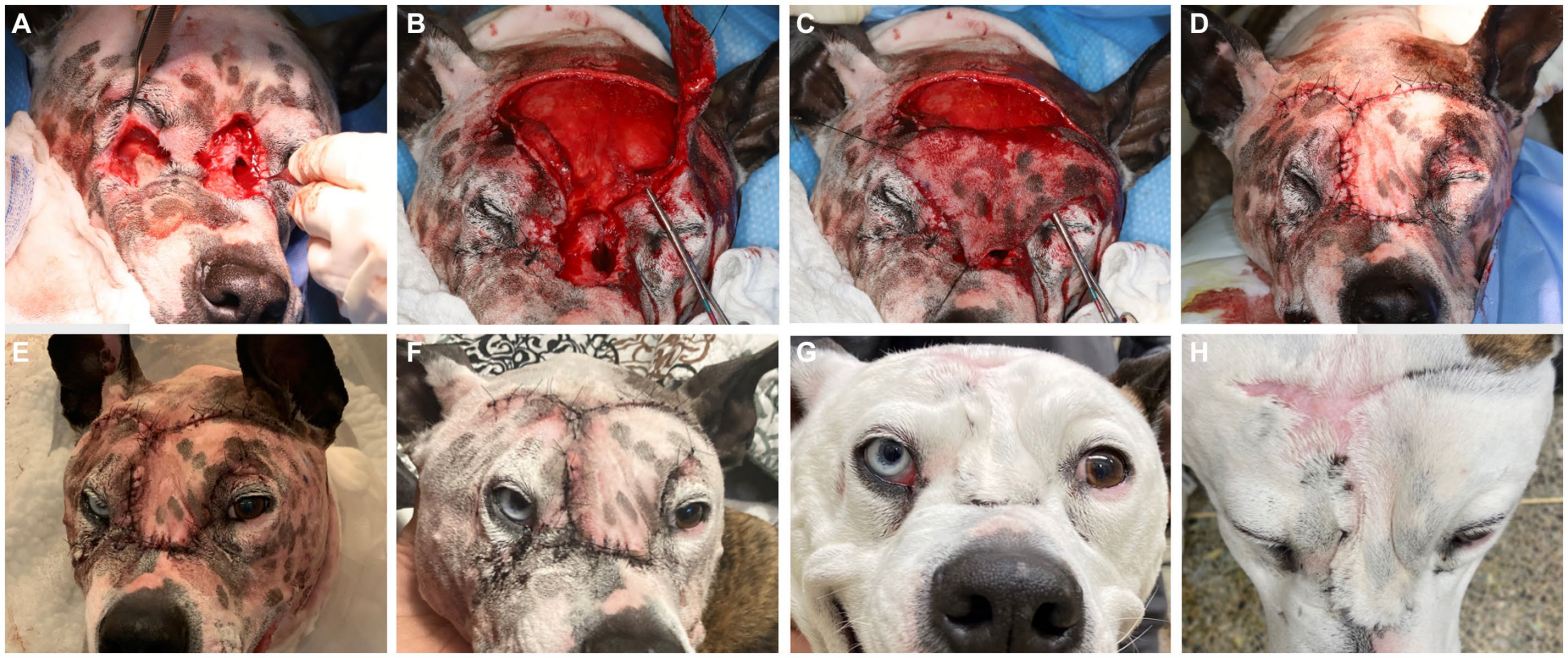
Lesions were elevated and debrided **(A)**. The smaller defects ventral to the left and right eye were closed prior to the elevation of the superficial temporal axial pattern flap **(B)**. Stay sutures were used to minimize iatrogenic trauma to the elevated flap to allow rotation into the defect **(C)**. Tension-free closure was achieved **(D)** and the patient was kept in hospital for close observation of flap vitality **(E)**, which was excellent. A 10 days post-surgical intervention showed again excellent flap vitality and clinically good healing **(F)**. At the 6 months recheck the flap had completely healed with mild fibrous tissue formation on the dorsal aspect **(G,H)**.

The diagnostic results were consistent with severe soft tissue infection and extensive osteolysis of the frontal, maxillary, and nasal regions, resulting in multiple intra- and extraoral draining tracts. The presence of foreign plant material likely complicated the healing process or was a part of the initiating process. It was elected to first minimize the infection before reconstructive surgery was attempted, by means of an extended antimicrobial therapy. The reconstructive phase was staged in two procedures. First, an extraoral repair was performed using advancement flaps for the smaller defects (Figure 5A) and a superficial temporal axial pattern flap (Figures 5B–D) to restore the large mid-facial draining tract. Superficial temporal axial pattern flap was performed as previously described (9–11). Briefly, the base of the flap was located at the level of the zygomatic arch. The width of the flap approximated the length of the zygomatic arch: The caudal orbital rim was the rostral border of the flap, and the caudal aspect of the zygomatic arch represents the caudal flap margin. The flap length was the mid-dorsal orbital rim of the opposite eye. The flap was elevated deep to the frontalis muscle to help preserve its blood supply. The flap was elevated from the distal aspect and advanced towards its base (11). Advancement flaps recruited adjacent lax tissue and moved linearly to fill in the defect. A combination of poliglecaprone 25 4-0 in a simple interrupted pattern for the deeper layers and Nylon 3-0 in a simple interrupted pattern for the skin was used to close the surgical sites. The dog was hospitalized for supportive care and close observation for 2 days to observe for initial flap vitality (Figure 5E). An Elizabethan collar was placed until the recheck evaluation. The owner reported that the dog was doing well at home, and a 10 days postoperative photograph was obtained through electronic communication (Figure 5F). At the 2 weeks postoperative recheck, in person, the flaps exhibited appropriate healing and the sutures were removed.

Eight weeks after the extraoral reconstruction, the dog underwent a second-stage procedure to address the intraoral draining tracts that communicated with the nasal passages and were considered extensive oronasal fistulas. A comprehensive oral health assessment and treatment, including a full mouth radiograph, was performed. No tooth extractions were required, and periodontal cleaning was performed before the closure of the defects. Large mucoperiosteal flaps were raised and undermined to allow for tension-free closure (Figures 2B,C).

Six months later, the dog was presented for a clinical recheck and a follow-up CT evaluation. The dog was reportedly doing well at home, and no dehiscence of the previously repaired oronasal and extraoral fistulas was noted (Figures 2D, 5G,H). Pre-anesthetic laboratory work revealed that all results were in reference ranges, and the previously noted lymphocytosis had resolved. The dog underwent general anesthesia for a repeat skull CT to assess the healing status. Clinical and CT imaging demonstrated advanced repair and remodeling processes, resolved small bone defects, and a substantial reduction in the size of the large central bone defect in the maxillary, nasal, and frontal bones consistent with clinically resolved infection, repair, and remodeling processes (Figures 3D–F).

## 3 Discussion

Chronic infection of the craniomaxillofacial region is a medical condition characterized by the inflammation of soft tissues and may involve infection of the bones in the skull, face, and/or maxilla region. An infection of the soft tissues and multiple osteolytic lesions including extensive draining tracts in the craniomaxillofacial area may be particularly challenging to manage due to the complex anatomy and potential ramifications in the ability to reconstruct causing minor to major disfigurements (3). The most reported causative pathogens for human craniomaxillofacial infections are *Staphylococcus aureus*, *streptococci*, and anaerobes (12, 13). The infection may develop from a variety of sources, such as dental infections, sinusitis, trauma, surgery, animal altercation, or the spread of infection from other areas of the body (3, 12, 13). In the case presented here, a combination of the aforementioned causes is believed to be contributing to the clinical presentation. First, unknown historical trauma may have created a lesion. Second, foreign plant material was retrieved on multiple occasions but may not have been able to penetrate the wounds if there was no wound to begin with. This plant material compromised the healing potential of the wounds and likely exacerbated or inoculated the infectious process. Third, the dog underwent multiple surgical procedures, effectively compromising the blood supply and delaying the healing with each intervention. Hence, a more aggressive repair technique using a large axial pattern flap was elected to source new blood supply to close the larger defect and aid in the healing process. Finally, the dog received a plethora of different antimicrobials. This may have contributed to the fact that a multi-drug resistant coagulase-negative *Staphylococcus* spp. was found on culture and sensitivity testing. Antimicrobial resistance represents one of the most important human and animal health-threatening issues worldwide (14).

Functional imaging of the craniofacial region is necessary for a complete treatment regimen in patients with acute or chronic infection (3, 12, 13). Computed tomography, cone beam CT, and magnetic resonance imaging (MRI) may be used for early detection of the condition (15). Bone scintigraphy is more accurate than CT when used in the detection of craniofacial osteomyelitis (15). CT may demonstrate bone erosion, lysis, or remodeling that may be confused with osteomyelitis versus a neoplastic process. Due to the chronicity of the condition in the dog presented here, it was elected to combine advanced imaging such as conventional contrast CT with endoscopy to visualize all possible lesions and obtain presentable samples for further analysis. Endoscopy was performed following CT imaging to avoid iatrogenic tissue changes from scope manipulations. A drawback of cone beam CT is its limitation in assessing soft tissue pathology due to the lack of contrast resolution and Hounsfield Units (HU) (16). Only when there is bone involvement, cone beam CT can be considered but would still be considered inferior to conventional CT (17).

Given the history, clinical presentation, and diagnostic imaging findings of the dog presented here, the condition was considered chronic. It was elected to perform a staged procedure to optimize the outcome. First, a long-term course of antimicrobials based on culture and sensitivity results was provided to minimize the bacterial burden. Second, an extraoral approach to reconstruction was initiated. The rationale of the authors was that any residual infected and necrotic tissue could continue to drain from the lowest point, as infectious disease processes tend to follow the path of least resistance (18). In this dog, the intraoral draining tracts were considered the lowest point among all the lesions and were therefore repaired at a later stage. It is likely that the draining tracts were fistulas (i.e., lined by epithelia), but we did not evaluate all tracts by histological means, and therefore, the term “draining tracts” was used. Reconstruction of large facial defects often requires the use of locoregional axial pattern flaps such as the *caudal auricular*, the *superficial temporal*, or the *angularis oris* myocutaneous axial pattern flaps (9–11). Small-to-moderate facial defects can be closed with local advancement or transposition skin flaps (10). The combination of a superficial temporal axial pattern flap for the large midfacial defect and local advancement flaps for the remaining defects achieved a successful closure of the lesions. After continuous drainage of infectious exudates through the intraoral defects had subsided and resolved, the oronasal defects were repaired with local mucosal advancement flaps (10). Clinical success was achieved based on healing of the intra- and extraoral surgical sites and confirmed by means of repeat CT imaging, which documented repaired and remodeled osseous lesions with intact overlying soft tissue.

## Data availability statement

The original contributions presented in the study are included in the article/supplementary material, further inquiries can be directed to the corresponding author.

## Ethics statement

Ethical review and approval was not required for the animal study because the study is retrospective in nature and included a clinical case, hence, it is exempt from IACUC requirements. The standard written informed consent was required for all procedures performed at the William R. Pritchard Veterinary Medical Teaching Hospital of the University of California, Davis was obtained from the owners.

## Author contributions

EW: Conceptualization, Writing - original draft. CK: Writing - review & editing. NV: Writing - review & editing. BA: Funding acquisition, Writing - review & editing.
